# Evaluation of Supercritical Extracts of Algae as Biostimulants of Plant Growth in Field Trials

**DOI:** 10.3389/fpls.2016.01591

**Published:** 2016-10-25

**Authors:** Izabela Michalak, Katarzyna Chojnacka, Agnieszka Dmytryk, Radosław Wilk, Mateusz Gramza, Edward Rój

**Affiliations:** ^1^Department of Advanced Material Technologies, Faculty of Chemistry, Wrocław University of Science and TechnologyWrocław, Poland; ^2^AGRECO LtdWrocław, Poland; ^3^Supercritical Extraction Department, New Chemical Syntheses InstitutePuławy, Poland

**Keywords:** algae, supercritical fluid extraction, biostimulant, field trials, winter wheat

## Abstract

The aim of the field trials was to determine the influence of supercritical algal extracts on the growth and development of winter wheat (variety *Akteur*). As a raw material for the supercritical fluid extraction, the biomass of microalga *Spirulina plantensis*, brown seaweed – *Ascophyllum nodosum* and Baltic green macroalgae was used. Forthial and Asahi SL constituted the reference products. It was found that the tested biostimulants did not influence statistically significantly the plant height, length of ear, and shank length. The ear number per m^2^ was the highest in the group where the Baltic macroalgae extract was applied in the dose 1.0 L/ha (statistically significant differences). Number of grains in ear (statistically significant differences) and shank length was the highest in the group treated with *Spirulina* at the dose 1.5 L/ha. In the group with *Ascophyllum* at the dose 1.0 L/ha, the highest length of ear was observed. The yield was comparable in all the experimental groups (lack of statistically significant differences). Among the tested supercritical extracts, the best results were obtained for *Spirulina* (1.5 L/ha). The mass of 1000 grains was the highest for extract from Baltic macroalgae and was 3.5% higher than for Asahi, 4.0% higher than for Forthial and 18.5% higher than for the control group (statistically significant differences). Future work is needed to fully characterize the chemical composition of the applied algal extracts. A special attention should be paid to the extracts obtained from Baltic algae because they are inexpensive source of naturally occurring bioactive compounds, which can be used in sustainable agriculture and horticulture.

## Introduction

Recently, there is increased interest in natural products that stimulate the growth of plants. A special attention has been paid to the raw material – the biomass of algae that is useful in the production of plant growth biostimulants (Calvo et al., 2014). The natural products obtained from algae constitute the subject of interest in agriculture with emphasis on its application in sustainable agriculture (Khan et al., 2009).

Algae have long been viewed a valuable source of food and traditional remedies. Over the centuries, various types of macroalgae, such as *Undaria* and *Laminaria* were grown and harvested in coastal areas. Another important commercial application of algae is the production of healthcare products and cosmetics, as well as the biochemical industry. Dietary supplements based on *Chlorella* and *Spirulina* are the most popular and successful commercial products from algae (Fradique et al., 2010; Moorhead et al., 2011; Enzing et al., 2014).

The beneficial, from the viewpoint of agricultural applications, properties of algae result from their living conditions – permanent abiotic and biotic stress. This caused that these organisms developed mechanisms that protect them from drought, salinity, changing light intensity, frost, colonization by bacteria or fungi. Resulting, algal cells contain bioactive compounds that are prospective in the protection of plants – carbohydrates, minerals, and trace elements, growth hormones (cytokinins, auxins and auxin-like compounds), betaines, sterols (Khan et al., 2009).

Since chemical synthesis of biologically active compounds is not profitable or difficult, the best source of these compounds are extracts obtained from algae. Algal extracts containing natural active compounds comprise a wide variety of structures and functions that provide excellent pool of molecules for the preparation of nutraceuticals, functional foods, food additives, and biological agrochemicals (Gil-Chávez et al., 2013; Jacob-Lopes et al., 2015).

By using different extraction techniques, it is possible to isolate these compounds in the process of extraction, to formulate products and find their direct applications. Among many extraction techniques (conventional liquid-liquid or solid–liquid extraction, pressurized-liquid extraction, subcritical and supercritical extractions, microwave- and ultrasound-assisted extractions), supercritical fluid extraction (SFE) is gaining increasing interest because the obtained extract is the concentrate of biologically active compounds in a solvent-free environment and is safe to plants (Gil-Chávez et al., 2013; Michalak and Chojnacka, 2014). These extraction techniques can be improved with biomass pretreatment steps (enzyme-and instant controlled pressure drop-assisted extractions) which improve the efficiency of extraction process (Gil-Chávez et al., 2013).

Upstream and downstream processing of supercritical algal extracts production was described by (Wilk and Chojnacka, 2015a,b). Supercritical carbon dioxide extraction was pointed out as a promising method for the production of algal-based biostimulants. High efficiency, full biodegradability, no phytotoxicity make biostimulants based on algal extracts promising plant protection products (Kim and Chojnacka, 2015).

Algae and their extracts can be used in crop management to reduce abiotic and biotic stresses (Sharma et al., 2014). They can act as chelators by the improvement of the utilization of mineral nutrients by plants and improvement of soil structure and aeration, which may stimulate root growth (Milton, 1964). As biostimulants of plant growth, algal extracts enhance seed germination, improve plant growth, yield, flower set and fruit production, as well as a post-harvest shelf life (Khan et al., 2009; Calvo et al., 2014). Because seaweed extracts are the inexpensive source of naturally occurring plant growth regulators they can be successfully used in sustainable agriculture and horticulture (Panda et al., 2012).

It is important to emphasize that algal extracts obtained by SFE have not been studied in the field experiments, so far. The results presented in the present paper are a continuation of the field trials conducted in the growing season 2013/2014 on winter wheat using supercritical *Spirulina* sp. extract in two rates: 1.20 and 1.8 L/ha (Chojnacka et al., 2014). The basis to conduct field trials were germination tests carried out on different species of plants using supercritical extracts: from the mixture of marine macroalgae from the Baltic Sea (species of *Polysiphonia, Ulva*, and *Cladophora*) and *Spirulina* sp. on cress (*Lepidium sativum*) (Dmytryk et al., 2014a,b) and from the mixture of Baltic macroalgae on garden cress and wheat (Michalak et al., 2016).

The objective of the present work was to evaluate the efficacy of biostimulants (different types of supercritical algae extracts) and their impact on the yield quantity and quality of winter wheat. As a raw material for the production of extracts was used the biomass of microalga *Spirulina platensis* and two macroalgae: the mixture of Baltic macroalgae (Poland, Sopot city) and *Ascophyllum nodosum* from Atlantic Ocean (France, Brittany).

## Materials and Methods

### Feedstock

The characteristics of commercially available *S. platensis* provided by WB Im-und Export W. Beringer & Co. GmbH. was described in the work of Chojnacka et al. (2014). Marine macroalgae were collected from the Baltic Sea directly from the water near Sopot beach (Poland). The detailed description of the harvesting of the biomass and pre-treatment before extraction process was described in the work of Michalak et al. (2016). Brown macroalga – *A. nodosum* was supplied by Laboratoires Goëmar SAS^1^. It was collected by hand from the Brittany coast (France) in June 2014 and stored in the frozen state. Before SFE, the biomass was defrosted in a fridge in the temperature ∼5°C. Then it was dried to the moisture 15% in the temperature 40°C. Finally, it was ground in the mill (Retsch SM 300) in order to obtain the fraction of the size 500 μm.

### Supercritical Extraction of the Biomass with CO_2_

Supercritical fluid extraction of all examined algal biomasses was performed in the New Chemical Syntheses Institute in Puławy – Supercritical Extraction Department (Poland). SFE of microalga *Spirulina* sp. was described in the work of Chojnacka et al. (2014) and of Baltic green macroalgae in the work of Michalak et al. (2016). In the case of brown macroalga *A. nodosum*, SFE was performed in the laboratory extractor with the volume – 1L. See Rój (2014) for further details. The following conditions: pressure 500 bar; temperature 50°C; load mass 257 g; CO_2_ passed through a bed of algal biomass 23.8 kg; CO_2_ consumption in relation to the initial mass of the plant sample was 100 kg CO_2_/1 kg of the load mass were used. The mass of the obtained liquid, dark brown extract and water was 30.9 g; extraction yield was 5.77%.

### Algal Extracts Tested in the Field Experiments

The following products were tested in field experiments on winter wheat, variety *Akteur* (**Table 1**) – growing season 2014/205. The choice of the application rates was based on the previous field experiments described by Chojnacka et al. (2014). The dose of the formulation containing supercritical algal extract was selected on the basis of the content of polyphenols in the applied reference product – Asahi SL. The concentration of polyphenols in the supercritical extract from *Spirulina* sp. was 3.0%. The prepared formulation in one liter contained 100 g of extract – the concentration of polyphenols was then equal to 0.3%. The content of polyphenols in this preparation (dose 1.20 L/ha) was the same as the content in the commercial product – Asahi SL (dose 0.60 L/ha) (Chojnacka et al., 2014).

**Table 1:** Products tested in field experiments on winter wheat.

Name	Rate, L/ha	Active substance
**Tested product – supercritical algal extracts**		
Baltic Sea algal extract	1.0	10% of Baltic Sea algal extract by weight
*Ascophyllum nodosum* extract	1.0	10% of *A. nodosum* extract by weight
*Spirulina platensis* extract	1.0	10% of *Spirulina* extract by weight
*S. platensis* extract	1.5	10% of *Spirulina* extract by weight
*S. platensis* extract	1.8	10% of *Spirulina* extract by weight
**Reference product**		
Control	Untreated	–
Forthial	1.0	N-NO _3_ – 6.2%; MgO – 9.0% Biologically active GA 142 filtrate from *A. nodosum* marine algae
Asahi SL	0.6	Bioactive compounds: sodium para-nitrophenolate – 0.3%, sodium ortho-nitrophenolate – 0.2%, sodium 5-nitroguaiacolate – 0.1%

In the present study, *Spirulina* extract was applied in three different rates: 1.0, 1.5, and 1.8 L/ha in order to examine if the extract dose stimulates the plant growth and development. It was the continuation of field experiments conducted in the growing season 2012/2013 on the winter wheat, variety *Tacitus*, when supercritical *Spirulina* extract was applied in two rates: 1.2 and 1.8 L/ha (Chojnacka et al., 2014).

The formulations for the field trials were prepared according to the data collected in **Table 2**. One liter of the final formulation of algal extract was diluted in 200 L of water and applied on one hectare (spray volume – 200 L/ha).

**Table 2:** The composition of formulations designed for field trial containing supercritical extracts of *S. platensis*, Baltic macroalgae, and *A. nodosum.*

Active substance	(% mas.)	Remarks
Supercritical algal extract	10.0	
Amphoteric emulsifier (Atlox 4915)	2.50	Croda Europe Ltd.
Non-ionic emulsifier (Atlas G-5000)	2.50	Croda Europe Ltd.
Potassium sorbate	0.010	C_6_H_7_KO_2_, POCH
Polyethylene glycol	2.50	POCH
B(III)	0.020	H_3_BO_3_, POCH
Cu(II)	0.050	CuSO_4_⋅5H_2_O, Acros Organics
Fe(II)	0.100	FeSO_4_⋅7H_2_O, Acros Organics
Mn(II)	0.050	MnSO_4_⋅H_2_O, POCH
Mo(VI)	1.00⋅10^-3^	(NH_4_)_6_Mo_7_O_24_⋅4H_2_O, POCH
Zn(II)	0.050	ZnSO_4_⋅7H_2_O, POCH
Demineralised water	fulfill to 100%	

### Field Experiments

The experiments were performed in accordance to the EPPO PP 1/144 (3), EPPO PP 1/135(4), EPPO PP 1/152 (4), and EPPO PP 1/181(4) guidelines. The trial site was Miechowice Oławskie, Lower Silesia, South-western Poland, GPS coordinates (N 50° 49′ 00,9″; E 17° 13′ 58,2″). The experiments were performed in randomized complete blocks in four replications (*N* = 4) for each tested product. The plot size was 20.0 m^2^ (2.0 m × 10.0 m). The soil had the following characteristics – soil type: loam, soil quality class: IIb, organic matter: 2.9%, soil pH: 6.4.

The sowing of winter wheat was on 3/10/2014 (the sowing density of winter wheat was 200 kg/ha), the harvesting on 30/07/2015 (BBCH 89: *fully ripe – grain hard, difficult to divide with thumbnail*). The tested products were applied twice: on 22/04/2015 (crop growth stage BBCH 31–32: *31 – first node at least 1 cm above tillering node; 32 – node 2 at least 2 cm above node 1*) and 3/06/2015 (crop growth stage BBCH 59–61: *59 – end of heading: inflorescence fully emerged; 61 – beginning of flowering: first anthers visible*). The average temperature and the total rainfall (mm) during the field experiments was as follows: April 9.9°C and 11.6 mm, May 13.8°C and 28.4 mm, June 17.4°C and 42.0 mm, July 21.1°C and 59.2 mm. The tested products were dosed to plants with the use of sprayer with a boom UP-02 (sprayer volume 6.0 L, nozzle ID: 8 nozzles TeeJet XR11002VS).

The trials were carried out with standard fertilization: 26/08/2014: CaCO_3_ (liming, CaO 50%, 2.2 t/ha), 02/10/2014: Polifoska 7 [NPK(S) 7-18-28(11), 150 kg/ha], 04/03/2015: ammonium nitrate (N 32%, 140 kg/ha), 14/03/2105: ammonium nitrate (N 32%, 140 kg/ha), 10/04/2015: ADOB (N-NO_3_ 2.7%, Cu 6.0%, 1.0 L/ha), 10/04/2015: magnesium sulfate (MgS 21–30, 5.0 kg/ha), 10/04/2015: OSD Mineral N 19.5%, 2.0 kg/ha, 22/04/2015: ammonium nitrate (N 32%, 140 kg/ha), 22/05/2015: ammonium nitrate (N 32%, 110 kg/ha). Full protection of plants was maintained (application of pesticides).

### Assessments Methods

The following parameters of plant growth were assessed. Before the first application of the tested products (22/04/2014; BBCH 31–32): crop height (cm) of 25 plants/plot and crop vigor – visual assessment on a 0–10 scale (5: control – optimal vigor, <5: worse vigor, >5: better vigor) were measured. Before the second application of the tested products (02/06/2015; BBCH 59–61) additionally phytotoxicity – visual assessment (0% – no phytotoxicity, 100% – plants totally destroyed) was performed.

Eight (30/04/2015; BBCH 8) and 21 (13/05/2015; BBCH 35–37) days after the first application of the tested products and 9 (12/06/2015; BBCH 64–67) and 22 (25/06/2015; BBCH 69–71) days after the second application: phytotoxicity and crop vigor were determined.

Before harvest (16/07/2015; BBCH 87: *hard dough – grain content solid. Fingernail impression held*) the following parameters were determined: crop height (cm), ear-bearing culms and barren culms number – 25 plants/plot, ear number per m^2^, grains in ear number – 25 ears/plot, length of ear (cm) – 25 ears/ plot, shank length (cm) – 25 ears/plot. Lodging assessment concerned the following observations: area lodged (%), intensity of lodging, phytotoxicity and crop vigor.

After the harvest (30/07/2015, BBCH 89), grain yield quantity based on a standard 15% moisture (t/ha) and mass of 1000 grains (g) were assessed.

### Statistical Methods

The results were elaborated statistically by *Statistica* ver. 10. Normality of distribution of experimental results was assessed by the Shapiro–Wilk test. On this basis, a statistical test was selected which was used to investigate the significance of the differences between the groups. For normal distribution, the differences between the groups were investigated with a one-way analysis of variance (ANOVA) using the Tukey test. If the distribution was not normal, the Kruskal–Wallis test was applied. Results were considered significantly different when *p* < 0.05.

## Results and Discussion

In the present paper, the effect of three supercritical algal extracts (obtained from *S. platensis, A. nodosum*, and Baltic macroalgae) and two reference materials (Forthial, Asahi SL) on the growth and development of winter wheat (variety *Akteur*) in the field trials was examined.

### Phytotoxicity, Plant Vigor, and Lodging Assessment

During the field experiments no phytotoxicity symptoms (0% – no phytotoxicity) were observed in the case of the application of all the tested products. Plant vigor was equal five for all products throughout the entire experimental period – till BBCH 87. The lodging assessment was performed in the crop growth stage BBCH 87. Area lodging (%) and lodging intensity was equal zero for all the preparations.

### Crop Height and the Number of Ear-Bearing Culm and Barren Culm

**FIGURE 1:**
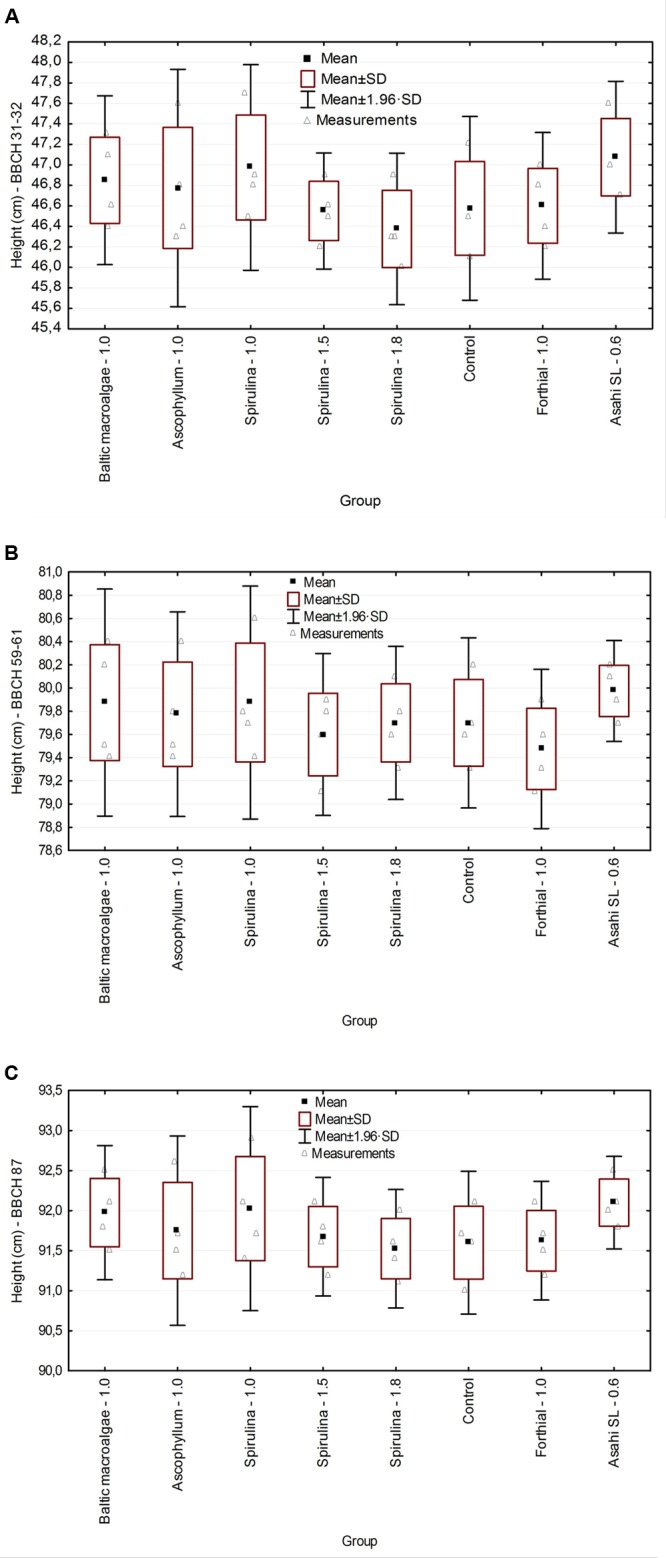
**Crop height (cm) at different BBCH crop growth stages: **(A)** 31–32, **(B)** 59–61, and **(C)** 87**.

**Figure 1** presents crop height at different BBCH crop growth stages. For all stages, the differences were not statistically different. The average height in the experimental and reference groups is presented in **Table 3**. The height of the winter wheat was comparable in all the groups. The examined algal extracts and reference products have no significant effect on plant growth. These results are in agreement with the results obtained by other authors. Matysiak et al. (2012) evaluated the effect of seaweed extracts from *Ecklonia maxima* (Kelpak SL) on the height of winter wheat, variety *Tonacja* in the field trials. It was found that the extract applied in autumn (BBCH 20) or in spring (BBCH 39) and twice application in the both terms in the rate 2 L/ha (soil irrigation) did not affect significantly the height of plants. However, the method of the application of the tested product – for example: foliar, soil application, or seed treatment can influence the plant growth (Khan et al., 2009; Michalak et al., 2016). In the work of Carvalho et al. (2014) it was found that the height of the wheat (*Triticum aestivum* cv. IAC 364) in a greenhouse depended on the method of the application of Acadian^®^ Marine Plant Extract produced from *A. nodosum*. Plants irrigated with *A. nodosum* extract (5 mL/L) were higher than the control (water) and when the seeds were previously treated with the extracts (0.1 mL of the *A. nodosum* extract on the 100 g of seeds), regardless of the evaluation period – at 14^th^, 28^th^, and 42^nd^ days after sowing.

**Table 3:** Crop height (cm) at different BBCH crop growth stages.

Group – rate (L/ha)	BBCH stage					
	31–32		59–61		87	
	Average (*N* = 4)	*SD*	Average (*N* = 4)	*SD*	Average (*N* = 4)	*SD*
Baltic macroalgae – 1.0	46.85	0.42	79.88	0.50	91.98	0.43
*Ascophyllum* – 1.0	46.78	0.59	79.78	0.45	91.75	0.60
*Spirulina* – 1.0	46.98	0.51	79.88	0.51	92.03	0.65
*Spirulina* – 1.5	46.55	0.29	79.60	0.36	91.68	0.38
*Spirulina* – 1.8	46.38	0.38	79.70	0.34	91.53	0.38
Control	46.58	0.46	79.70	0.37	91.60	0.45
Forthial – 1.0	46.60	0.37	79.48	0.35	91.63	0.38
Asahi SL – 0.6	47.08	0.38	79.98	0.22	92.10	0.29

In the present study it was shown that the average ear-bearing culms number (25 plants/plot) in BBCH 87 was 4.1 in the tested groups with the exception of Baltic macroalgae and *Ascophyllum* for which it was 4.0. The average barren culms number was 0.1 for the tested groups with the exception of Baltic macroalgae and *Spirulina* – 1.5 for which it was 0. The effect of algal extracts on these parameters of wheat was not studied in the literature. Therefore the comparison is not possible.

### Effect of Biostimulants on the Ear Number of Winter Wheat and the Number of Grains in Ear

**FIGURE 2:**
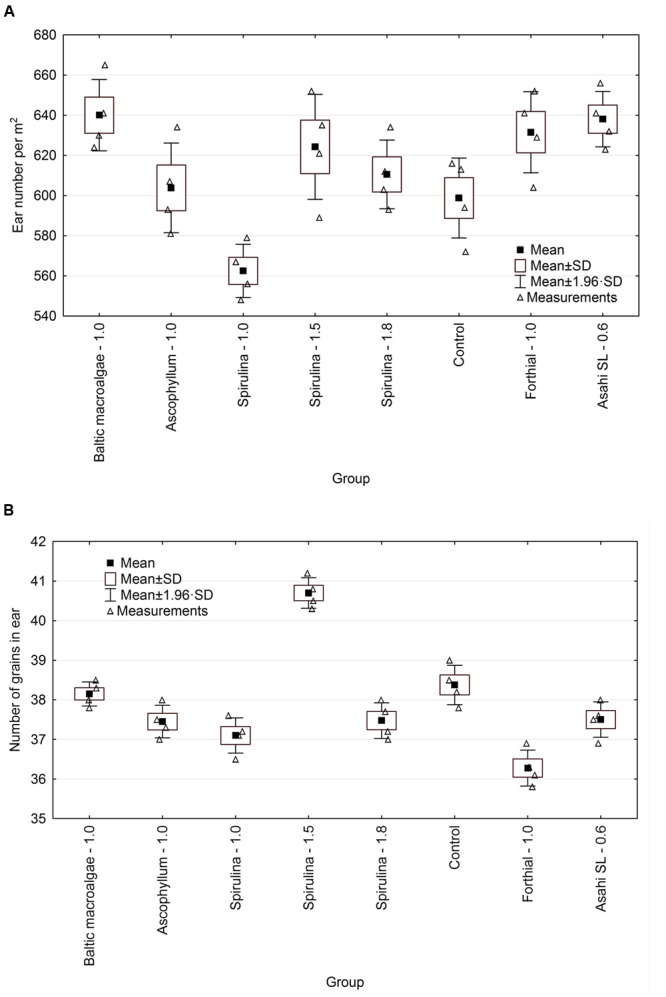
**Effect of biostimulants on various parameters of winter wheat – **(A)** ear number per m^2^, **(B)** number of grains in ear**.

The effect of the tested biostimulants on the ear number per m^2^ and number of winter wheat grains in ear is presented in **Table 4**. Statistically significant differences between tested products were observed for both measured parameters (**Figure 2**).

**Table 4:** Effect of biostimulants on various parameters of winter wheat.

Group – rate (L/ha)	Ear number per m^2^		Number of grains in ear		Length of ear (cm)		Shank length (cm)	
	Average (*N* = 4)	*SD*	Average (*N* = 4)	*SD*	Average (*N* = 4)	*SD*	Average (*N* = 4)	*SD*
Baltic macroalgae – 1.0	640.00^a^	18.09	38.15^abc^	0.31	8.60	0.26	11.93	0.17
*Ascophyllum* – 1.0	603.75	22.79	37.45^de^	0.42	8.73	0.39	11.93	0.22
*Spirulina* – 1.0	562.50^abcde^	13.48	37.10^afg^	0.45	8.55	0.29	12.28	0.43
*Spirulina* – 1.5	624.25^b^	26.70	40.70^bdfhijk^	0.39	8.68	0.41	12.55	0.13
*Spirulina* – 1.8	610.50^c^	17.48	37.48^hk^	0.46	8.55	0.37	12.15	0.25
Control	598.75	20.32	38.38^gil^	0.51	8.68	0.24	11.98	0.33
Forthial – 1.0	631.50^d^	20.60	36.28^cejklm^	0.46	8.30	0.36	12.05	0.42
Asahi SL – 0.6	638.00^e^	14.07	37.50^km^	0.45	8.58	0.38	12.20	0.36

*a, b, c… – statistically significant differences (*p* < 0.05) between examined groups.*

For ear number per m^2^, the best results were obtained for the extract from Baltic macroalgae and the weakest for *Spirulina* extract – 1.0. The average ear number for group with Baltic macroalgae was 13.8% higher than for *Spirulina* extract – 1.0 (statistically significant difference). Statistically significant differences were also noted between this group and *Spirulina* – 1.5, *Spirulina* – 1.8, Forthial and Asahi SL. Only for *Spirulina* – 1.0, the ear number per m^2^ was lower than in the control group – untreated.

This result is in agreement with the data obtained in the growing season 2012/2013 when *Spirulina* extract was applied on wheat at two doses: 1.2 and 1.8 L/ha (Chojnacka et al., 2014). The ear number per m^2^ for 1.2 L/ha was 3.0% higher than for 1.8 L/ha and 11% higher than for the control group (untreated, without fertilization). In this growing season, this parameter was 11% higher for *Spirulina* – 1.5 than for *Spirulina* – 1.0, 2.3% than for *Spirulina* – 1.8 and 4.3 than for the control group (untreated, with standard fertilization). In the work of Carvalho et al. (2014), it was also found that the number of ear of wheat (*T. aestivum* cv. IAC 364) was significantly affected after soil irrigation with *A. nodosum* extract (Acadian^®^– 5 mL/L) when compared with the control group (water).

In the case of the number of grains in ear, the best results were obtained for extract from *S. platensis* applied in the rate 1.5 L/ha. For this group, the average number of grains in ear was 6.0% higher when compared to the control group, 6.7% for Baltic macroalgae, 8.5% for Asahi SL, 8.6% for *Spirulina* – 1.8 L/ha, 8.7% for *Ascophyllum*, 9.7% for *Spirulina* – 1.0 L/ha and 12.2% for Forthial. Only biostimulant – extract from *Spirulina* (1.5 L/ha) showed better results than the control group – untreated. Matysiak et al. (2012) reported that the tested product – Kelpak SL at a dose 2 L/ha did not have impact on the number grain in ear.

### Yield Parameters

**FIGURE 3:**
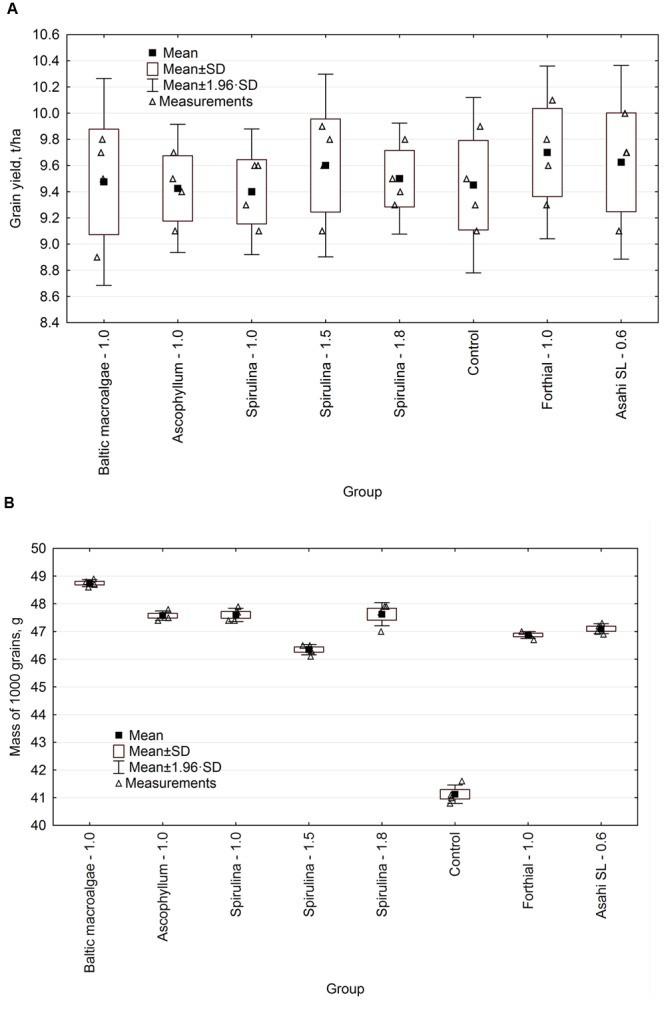
**Yield **(A)** (yield calculated at 15% moisture) and yield parameter – mass of 1000 grains (B)**.

The effect of the tested biostimulants on the yield parameters of grain and mass of 1000 grains is presented in **Figure 3**. The grain yield was comparable in the all tested groups – there were no statistically significant differences. These differences were noted for the mass of 1000 grains (**Table 5**). The best grain yield was obtained for Forthial, the weakest for *Spirulina* extract – 1.0. The difference was 300 kg from one hectare, however, this difference was not statistically significant. For the groups: Baltic macroalgae – 1.0, *Spirulina* – 1.5, *Spirulina* – 1.8, Forthial – 1.0, Asahi SL – 0.6, the grain yield was higher than in the control group by 25, 150, 50, 250, and 175 kg, respectively, from one hectare. Among supercritical extracts, *Spirulina* – 1.5 L/ha provided the highest yield.

**Table 5:** Winter wheat yield and yield parameter.

Group – rate (L/ha)	Grain yield, t/ha		Mass of 1000 grains, g	
	Average (*N* = 4)	*SD*	Average (*N* = 4)	*SD*
Baltic macroalgae – 1.0	9.48	0.40	48.75^abcdefg^	0.13
*Ascophyllum* – 1.0	9.43	0.25	47.58^ahij^	0.17
*Spirulina* – 1.0	9.40	0.24	47.60^bklm^	0.24
*Spirulina* – 1.5	9.60	0.36	46.35^chknop^	0.19
*Spirulina* – 1.8	9.50	0.22	47.63^dnrs^	0.43
Control	9.45	0.34	41.13^eilortu^	0.34
Forthial – 1.0	9.70	0.34	46.88^fjmst^	0.13
Asahi SL – 0.6	9.63	0.38	47.10^gpu^	0.18

*a, b, c… – statistically significant differences (*p* < 0.05) between examined groups.*

In the case of the mass of 1000 grains, this parameter was the highest for the extract from Baltic macroalgae, the lowest for the control group (18.5% more than for control group – statistically significant difference). These results do not coincide with the yield data. In the work of Carvalho et al. (2014), this parameter was also examined. The dry mass of 100 grains in the group of wheat (*T. aestivum* cv. IAC 364) treated with *A. nodosum* (5 mL/L) was only 8% higher than in the control group (water). Shaaban (2001) evaluated the effect of different concentration of water extract of microalga *Chlorella vulgaris* (25, 50, 75, and 100%) on the yield of wheat (*T. aestivum* L. cv. Giza 69) in the greenhouse. It was found that dry mass of the shoots treated previously with 50% algal extract led to 81.4% dry weight increase when compared with control (water) – difference significant statistically. The increase of the dry biomass can be a reflection of the increase of nutrient uptake and concentration of algal extract. This concentration of algal extract led also to the increase of weight of 100 grains by more than 40% when compared with control.

### Effect of the Dose of *Spirulina* Extract on Winter Wheat Growth

*Spirulina* extract was applied in three different rates: 1.0, 1.5, and 1.8 L/ha in order to examine if the extract dose stimulates the plant growth and development. It was shown that the lower rate of the biostimulant, the higher plant height. For BBCH stages 31–32 and 87, the order was as follows: 1.0 > 1.5 > 1.8. Only for BBCH stage 59–61, the order was different: 1.0 > 1.8 > 1.5. In the case of ear number per m^2^ and number of grains in ear, the order was the same: 1.5 > 1.8 > 1.0. The highest yield was obtained for 1.5 L/ha – the yield was higher by 100 kg from one hectare when compared to 1.8 L/ha and by 200 kg when compared to 1.0 L/ha (1.5 > 1.8 > 1.0). These results do not correspond with the data concerning the mass of 1000 grains (1.8 > 1.0 > 1.5). Taking into account the yield, which is the most important parameter that shows the effect of the tested product on plant growth, the *Spirulina* extract applied in the rate 1.5 L/ha was the most efficient.

In the previous field trials, performed in the growing season 2012/2013 on winter wheat (variety *Tacitus*), it was found that the yield was also higher for lower concentration of supercritical extract from *S. platensis* (1.2 L/ha – 5.58 ± 0.5 t/ha) than for the higher dose (1.8 L/ha – 5.10 ± 0.63 t/ha). The yield in this growing season was much lower than in the current one. The reason is the used standard fertilization in 2014/2015. In 2012/2013 only the tested biostimulants were applied. For the applied rate of *Spirulina* extract – 1.8 L/ha, the yield in 2015 was 83% higher (9.5 t/ha) than in 2013 (5.2 t/ha) (Chojnacka et al., 2014). The obtained results showed that algal extracts can be used as the supplementation of the standard fertilization which is necessary to obtain satisfactory yield.

Previously, it was also confirmed in the germination tests that the higher concentration of supercritical extract from Baltic macroalgae (27.6 mg/L when compared with 13.8 mg/L) had inhibitory effect on the length and weight of wheat, which is a typical effect of phytohormone activity (Michalak et al., 2016). The modern concept of plant hormones suggests that bioactive compounds can influence physiological process in plants at low concentrations and can inhibit at higher concentration (Davies, 2004). Other example was presented by Kumar and Sahoo (2011) who examined the effect of water extract obtained from *Sargassum wightii* by boiling in distilled water on the seed germination, growth and yield of wheat (*T. aestivum* var. Pusa Gold). In the germination tests on Petri plates, different concentrations of the extract were applied: 5, 10, 20, 30, 40, 50, and 100%. All growth and yield parameters were found to be highest for the 20% concentration of algal extract. The increased growth and yield at low concentrations may be due to the presence of some growth regulators, as well as micro- and macronutrients (Challen and Hemingway, 1965) and vitamins Shaaban (2001). They may improve a nutrient assimilation and solute translocation from leaves to grains which led to significant increase in yield and grain weight (Shaaban, 2001).

Among the mentioned bioactive compounds, a special attention should be paid to the phytohormones. For example, auxins play a crucial role in induction of elongation growth, initiation of root formation, etc.; cytokinins in control of cell division, bud development, development of the leaf blade; gibberellins in stem elongation; initiation of seed germination (Tarakhovshaya et al., 2007).

Algae are known to contain plant hormones (Tarakhovshaya et al., 2007; Khan et al., 2009). However, their concentration is rarely studied especially in algal extracts obtained by SFE. In our previous studies it was shown that supercritical extract obtained from Baltic seaweeds contained plant hormones such as: phenylacetic acid (PAA) from auxin group and 6-benzylaminopurine (6-BA) from cytokinin group (Michalak et al., 2016). In the literature it was confirmed that extracts obtained by other extraction techniques from *Spirulina* sp. and *A. nodosum* also contain plant hormones. In the work of Amin et al. (2009) it was found that the *Spirulina* aqueous extract contained phytohormones such as: indole-3-acetic acid (IAA), gibberellic acid (GA), benzyladenine (BA), abscisic acid (ABA), jasmonic acid (JA), methyl jasmonic acid (MeJA) in relatively high volume. Kingman and Moore (1982) identified the purine adenine, the auxin IAA and abscisic acid (ABA) in crude extracts of *A. nodosum*.

The effect of algal extract on the plant growth depends on many factors, for example: dose, method and time of application, selected cultivar (Carvalho et al., 2014). In the work of Anisimov and Chaikina (2014) it was also confirmed that extracts obtained from various algae have a different effectiveness. This can be due to differences in the composition and the content of plant growth regulators in extracts of the different seaweeds.

### Summary of the Field Trails

There is a growing interest in the application of natural products in agriculture that will increase the crop productivity without causing further environmental degradation (e.g., eutrophication, soil infertility, and biodiversity loss). This results from the rapidly growing world population and the need to significantly increase food production (Arioli et al., 2015; Garcia-Gonzalez and Sommerfeld, 2016). The use of algal extracts, proposed in the present study, produced by SFE, can constitute the solution to the problem. Moreover, supercritical algal extracts are safe for the plants, as well as plant-derived products for human and animals. This is possible due to application of CO_2_ as a solvent in the extraction process instead of organic solvents. Algal based products are environment-friendly and non-toxic (Michalak and Chojnacka, 2014). There is no problem with residues, toxicity, ecotoxicology, fate and behavior in the environment, as in the case of synthetic plant protection products. Resulting, because of that it is expected that simplified route in the registration of the products of natural origin will be implemented in the obligatory role. Moreover, the species of algae used in the present study are recognized as safe (Enzing et al., 2014; Arioli et al., 2015). Using algal extracts as biostimulants of plant growth is a natural solution that guarantee the growth of food vegetables/fruits without any chemical residues. These natural products provide improved crop quality with full respect for human health and the environment (du Jardin, 2015; Povero et al., 2016).

In the present paper it was shown that no phytotoxic symptoms were observed on the crop of winter wheat (cv. *Akteur*) neither from the tested biostimulants: Baltic Sea algal extract, *A. nodosum* extract, *Spirulina* extract, nor from the reference products Asahi SL and Forthial. Plant vigor was similar in all the treated and the untreated plots. There was no effect of treatments on plant vigor. Crop height was similar in all the treated and the untreated plots. There were no significant differences in ear-bearing culms’ and barren culms’ number between the treated and the untreated plots. Significantly higher ear number per m^2^ was assessed on the plots treated with Baltic Sea algal extract and reference product Asahi SL. Lower ear number per m^2^ was noted on the plots with reference product Forthial and tested products *Spirulina* extract (1.5 L/ha; 1.8 L/ha). Significantly lower ear number per m^2^ was recorded on the plots with *A. nodosum* extract and on the untreated plots. Significantly the lowest ear number per m^2^ was assessed on the plots treated with *Spirulina* extract (1.0 L/ha). Significantly higher number of grains in ear was assessed on the plots with tested product *Spirulina* extract (1.5 L/ha). Lower grains number was noted on the untreated plots and plots treated with Baltic Sea algal extract. Significantly lower number of grains in ear was recorded on the plots with the reference product Forthial. There were no significant differences in ear length and shank length between the treated and the untreated plots. No lodging of plants was assessed on all the plots. Significantly higher mass of 1000 grains of winter wheat was assessed on the plots treated with Baltic Sea algal extract. Lower mass was noted on the plots with tested products: *A. nodosum* extract and *Spirulina* extract (1.0 and 1.8 L/ha) and reference products Asahi SL and Forthial. Significantly the lowest mass was recorded on the untreated plots and plots with tested product *Spirulina* extract (1.5 L/ha). There were no significant differences in the yield between the treated and the untreated plots.

The results of the present study showed that the formulations containing supercritical algal extracts showed similar biostimulant properties as products available on the market. However, they constitute the natural source of biologically active compounds, they were not produced in the chemical synthesis. Therefore, they can be treated as environmentally friendly. The results showed that this is an interesting direction of research. In future, more field experiments with different doses and different algae species should be conducted.

## Author Contributions

IM: Substantial contributions to the conception, design of the work; the acquisition, analysis, interpretation of data for the work; drafting the work; final approval of the version to be published. Agreement to be accountable for all aspects of the work. KC: Substantial contributions to the conception, design of the work; analysis of data for the work; revising the work critically for important intellectual content; final approval of the version to be published. AD: Substantial contributions to the conception of the work; or the acquisition of data for the work; drafting the work; final approval of the version to be published. RW: Substantial contributions to the conception, design of the work; or the acquisition of data for the work; drafting the work; final approval of the version to be published. MG: Substantial contributions to the conception, design of the work; Drafting the work; Final approval of the version to be published. ER: Substantial contributions to the conception, design of the work; the acquisition, analysis, interpretation of data for the work; revising the work critically for important intellectual content; final approval of the version to be published.

## Conflict of Interest Statement

The authors declare that the research was conducted in the absence of any commercial or financial relationships that could be construed as a potential conflict of interest.

## Footnotes

1 www.goemar.com
